# Structure-based analyses of neutralization antibodies interacting with naturally occurring SARS-CoV-2 RBD variants

**DOI:** 10.1038/s41422-021-00554-1

**Published:** 2021-09-03

**Authors:** Hua Xu, Bo Wang, Tian-Ning Zhao, Zi-Teng Liang, Tian-Bo Peng, Xiao-Hui Song, Jia-Jing Wu, You-Chun Wang, Xiao-Dong Su

**Affiliations:** 1 State Key Laboratory of Protein and Plant Gene Research, School of Life Sciences, and Biomedical Pioneering Innovation Center (BIOPIC), Peking University, Beijing, China; 2 Division of HIV/AIDS and Sex-transmitted Virus Vaccines, Institute for Biological Product Control, National Institutes for Food and Drug Control (NIFDC) and WHO Collaborating Center for Standardization and Evaluation of Biologicals, Beijing, China

**Keywords:** Nanocrystallography, Molecular biology

Dear Editor,

SARS-CoV-2 variants are developing rapidly among COVID-19 patients, likely resulting in higher transmissibility at the population level.^1–5^ Mutations in the spike proteins (S proteins) of these variants are supposed to be related with receptor binding and virus invasion. Examples of these prevalent variants include the B.1.1.7 lineage that emerged in the United Kingdom (UK), the B.1.351 lineage (also termed 501Y.V2) in South Africa (SA), and the P.1 and P.2 lineages in Brazil, etc. Many S protein alterations, especially in the receptor binding domain (RBD), characterize these variants, e.g., the N501Y mutation in B.1.351 and the K417N (or T)/E484K/N501Y co-mutation in the SA and Brazil variant.^2,3,5^ The RBD is responsible for interacting with mammalian receptor angiotensin-converting enzyme 2 (ACE2) to mediate the viral infection of host cells. It is also concentrated with epitopes for neutralizing antibodies (NAbs), thus playing a vital role in the study of prophylactics and therapeutics for COVID-19.^6–10^ Whether those RBD mutations may alter virus–host cell interactions and gain resistance to NAbs needs to be addressed.

From the public data, we have analyzed the mutational and co-mutational sites in the SARS-CoV-2 RBD (Supplementary information, Fig. S1). Among the mutations, the N501Y mutant, firstly identified in the UK,^2^ was the most abundant, followed by S477N, N439K, L452R, E484K, K417N (Supplementary information, Fig. S1a). The K417N/E484K/N501Y ternary mutation was the most abundant among co-mutations in the SA lineage^3^ (Supplementary information, Fig. S1b). Those common RBD mutations were highly transmissible and quickly spread to other countries (Supplementary information, Fig. S2).

Most published human NAbs have been isolated from convalescent patients, and the RBD of trimeric S protein is the major target.^7–10^ These potent NAbs showed great therapeutic and prophylactic efficacy, e.g., BD-368-2, which could bind to both “up” and “down” RBDs.^7,11^ Furthermore, the VH3-53/3-66 class-derived public antibodies were the most prevalent NAbs, and were identified in many COVID-19 patients worldwide.^7,9^

To assess the impacts of mutations on potent NAbs, we have used structural and biochemical approaches to systematically compare the interactions between several previously identified NAbs and major naturally occurring RBD mutations. By solving the crystal structures of fragment antigen-binding (Fabs) regions in complex with the RBD or its mutants, we reveal the affinity diversities of these antibodies to different mutations. Subsequently, we found that the VH3-53/3-66 class-derived public antibodies largely remain effective against most of the RBD variants studied.

We first solved the crystal structures of the BD-503 Fab in complex with the RBD, RBD-S477N, RBD-E484K, RBD-N501Y and RBD-501Y.V2 (Fig. 1a; Supplementary information, Table S1). BD-503 belongs to the VH3-53/3-66 public antibody class, and showed a similar binding mode to those of other antibodies in the same germline-based public antibody class (Fig. 1a; Supplementary information, Fig. S3a). The binding sites of these antibodies to the RBD would completely overlap those of ACE2 and block SARS-CoV-2 receptor binding, thus preventing viral infection (Fig. 1b). Mutations in the RBD would differently affect the epitopes of antibodies (Fig. 1c). The VH3-53/3-66 antibodies usually have five regions that interact with the RBD: CDRH1, CDRH2, and CDRH3 in the heavy chain and CDRL1 and CDRL3 in the light chain (Fig. 1c–g; Supplementary information, Fig. S3b). The S477N mutation barely interacted with BD-503, and the serine at position 477 did not directly contact the antibody (Fig. 1c–g). However, the natural K417N, E484K and N501Y mutations changed the binding sites of BD-503 (Fig. 1c and h–j). The K417 was not directly interacting with BD-503 while the K417N can be recognized by Y33 in the heavy chain (Fig. 1h). The E484K mutation broke the interaction between E484 and Y102 in the heavy chain of BD-503 (Fig. 1i). The naturally occurring N501Y mutation disrupted interactions of RBD N501 to S28 and Y92 in the light chain of BD-503, and also caused a slight shift to break the contact of RBD G502 to Q27, G496 to Y92 and Y449 to S30 in that chain of BD-503 (Fig. 1j). These changes may account for the reduced interaction affinities of BD-503 to RBD-E484K, RBD-N501Y and RBD-501Y.V2.

**Fig. 1:**
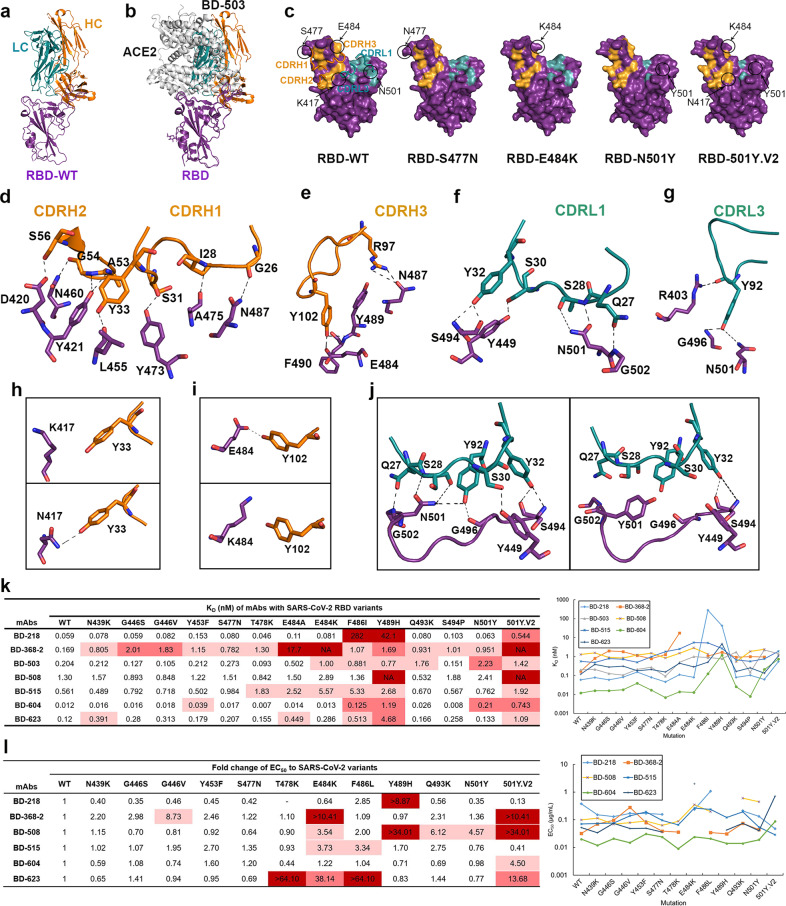
Interaction of SARS-CoV-2 RBD variants with neutralizing antibodies. **a** Crystal structure of BD-503 in complex with RBD. **b** BD-503 clashed with ACE2 binding to SARS-CoV-2 RBD, and therefore interfered with the interaction between RBD and ACE2. **c** Epitopes of BD-503 with SARS-CoV-2 RBD, RBD-S477N, RBD-E484K, RBD-N501Y and RBD-501Y.V2. CDRH1, CDRH2, CDRH3, CDRL1, and CDRL3 were involved in the interaction. The RBD is shown in a surface view. **d**–**g** Detailed contacts between BD-503 and SARS-CoV-2 RBD. **h** Y33 in the heavy chain of BD-503 can recognize K417N mutation site in RBD. **i** The E484K mutation in RBD broke the interaction between N484 and Y102 in the heavy chain of BD-503. **j** The N501Y mutation in RBD disrupted the interaction between N501 and BD-503 and broke the contact of G502 in RBD and Q27 in BD-503 light chain, G496 in RBD and Y92 in BD-503 light chain, Y449 in RBD and S30 in BD-503 light chain. **k** *K*_D_s of neutralizing antibodies binding to SARS-CoV-2 RBD and to naturally occurring variants as measured by SPR. Red indicates major fold-change larger than 100-fold. Rose indicates fold-change between 10- and 100-fold. Pink indicates minor fold-change between 3- and 10-fold. **l** EC_50_s and fold-changes of NAbs against pseudovirus carrying SARS-CoV-2 RBD mutations. Red indicates major fold-change that cannot be obtained from the assay. Rose indicates fold-change between 10- and 100-fold. Pink indicates minor fold-change between 3- and 10-fold.

We continued to investigate the impacts of the naturally occurring RBD mutations on potent NAbs by using surface plasmon resonance (SPR) by comparing the interactions between several previously identified NAbs with RBD variants (Fig. 1k; Supplementary information, Figs. S4–S6). We found that BD-368-2, which had shown best therapeutic and prophylactic efficacy compared to other potent NAbs,^7,11^ did not interact with variants containing the E484K mutation (Fig. 1k), which is highly abundant in SA and Brazil variant lineages (Supplementary information, Figs. S1, S2e, f). On the other hand, another NAb, BD-218, showed much greater affinity to most of the naturally occurring mutations, except for F486I and Y489H, suggesting that BD-218 requires these two residues to recognize the RBD (Fig. 1k; Supplementary information, Fig. S6). Fortunately, F486 and Y489 are not common RBD mutation sites (Supplementary information, Fig. S1a). We thus conclude that BD-218 could serve as a replacement for BD-368-2 in COVID-19 treatment.

The other five potent NAbs (i.e., BD-503, BD-508, BD-515, BD-604, and BD-623) belong to the VH3-53/3-66 public antibody class and possess a high degree of identity among their heavy chains (Supplementary information, Fig. S7). However, they bound differently to the RBD, with *K*_D_s from 1.3 nanomoles to 12 picomoles (Fig. 1k; Supplementary information, Fig. S4). The obvious affinity differences among those germline-based public antibodies may result from their light chains, since their heavy chains had similar epitopes (Supplementary information, Fig. S3b). Our data showed that these public antibodies all had high affinity to the majority of the naturally occurring RBD variants, except for BD-508, which had the lowest affinity, and could not bind to RBD-Y489H and RBD-501Y.V2 (Fig. 1k; Supplementary information, Figs. S4–S6). Our data showed that Y489H and RBD-501Y.V2 mutations caused more than a three-fold reduction of binding affinity to all five VH3-53/3-66 antibodies, and thereinto, BD-604 binds to RBD-501Y.V2 with a sixty-fold reduction in affinity and binds to RBD-Y489H with a nearly a hundred-fold reduction (Fig. 1k). BD-623 binds to RBD-Y489H with a nearly forty-fold reduction in affinity (Fig. 1k). Besides, the E484K, F486I and N501Y also showed decreased affinities to some antibodies, and BD-503 has a ten-fold reduction in affinity when binding to RBD-N501Y, lower than RBD-501Y.V2, and has a five-fold reduction binding to RBD-E484K (Fig. 1h–k). From the SPR data, the germline-based antibodies remained *K*_D_s of at least 5.57 nanomole to all the RBD variants (Fig. 1k; Supplementary information, Figs. S4–S6). Our results thus explained that the VH3-53/3-66-derived public antibodies can bind to many of the current mutants, consistent with the ongoing tendency that wild-type S protein-based vaccines do protect us from being infected by SARS-CoV-2 RBD mutants.

Epitopes of the VH3-53/3-66-derived antibodies have highly conserved heavy chain-binding sites that correlate to their high VH sequence identities (Supplementary information, Figs. S3b and S7). Epitopes recognized by the light chains of these public antibodies are distinct (Supplementary information, Fig. S3b). The RBD residues R403, D420, Y421, L455, Y473, A475, and N487 are most common binding sites for VH3-53/3-66-derived public antibodies that are recognized by almost all members in this class.^9–11^ Besides, the Y489, G502 and Y505 residues in the RBD can also be recognized by most VH3-53/3-66 antibodies. Another two residues, K417 and Y453, also commonly contact VH domains (CC12.1 and CC12.3), VL domains (BD-604, COVOX-150, and COVOX-158) or both (BD-236, CC12.1, and COVOX-158) (Supplementary information, Fig. S3b, c). These common RBD-binding sites that contact the VH3-53/3-66 antibodies, especially the residues recognized by VH domains, are essential for the VH3-53/3-66 public antibodies to recognize SARS-CoV-2. From the structure and SPR data, we found that the F486I, Y489H and 501Y.V2 mutations obviously affected the affinity to most VH3-53/3-66 antibodies (Fig. 1h–k), arousing public alert to the mutations on these residues.

To evaluate the ability of our antibodies to protect against SARS-CoV-2 variants, we performed neutralization assays using pseudoviruses expressing wild-type and different variants of the S protein (Fig. 1l; Supplementary information, Fig. S8). The results further proved that BD-368-2 completely lost its neutralizing activity against variants containing E484K mutation, while BD-218 performed well and even had higher EC_50_s to most of the variants except F486L and Y489H mutations; these two sites were demonstrated to be critical for interaction (Fig. 1k, l; Supplementary information, Fig. S8). Most of the VH3-53/3-66 class antibodies still have high neutralizing efficacy, especially the BD-604, whose EC_50_s were lower than 0.1 µg/mL to all the variants (Fig. 1l; Supplementary information, Fig. S8). The Y489H and 501Y.V2 variants escaped from the neutralization of BD-508, which is consistent with the affinity data, and the E484K, Q493K and N501Y mutations also had an impact on the neutralization activity of BD-508 (Fig. 1l; Supplementary information, Fig. S8). BD-515 was only slightly affected by E484K and F486L mutations (Fig. 1l). Unexpectedly, BD-623 lost neutralizing efficacy against T478K, E484K and F486L variants, and also showed a more than ten-fold reduction in EC_50_ to 501Y.V2 variant (Fig. 1l; Supplementary information, Fig. S8). Except these, the VH3-53/3-66-derived antibodies exhibited high neutralizing efficacy to most of the S protein variants.

In conclusion, we evaluated the binding affinity of several previously identified NAbs to SARS-CoV-2 RBD mutations to assess the impacts of those mutations on potent NAbs. We found that BD-368-2, previously shown with the best therapeutic and prophylactic efficacies against SARS-CoV-2,^7,11^ may not be effective in treating many of the SARS-CoV-2 variants. Among the NAbs we investigated, BD-218 and BD-604 showed significantly higher affinities to the variants, suggesting that those two antibodies are highly potent therapeutic candidates against COVID-19. Furthermore, most of the VH3-53/3-66-derived public antibodies maintained high affinity to most RBD variants, which is correlated well with their highly conserved RBD epitopes that were recognized by their highly conserved VH domains. Finally, we have performed pseudovirus neutralization assays to confirm that these germline-based antibodies kept high neutralizing/protecting efficacy against most of the S protein variants. Therefore, this common class of germline-based antibodies can still protect host cells from infection by emerging SARS-CoV-2 RBD variants, thus providing foundations for further design and development of antibody therapies against COVID-19.

## Supplementary information

Supplementary Information

## Data Availability

The crystal structures of BD-503 Fab in complex with RBD, RBD-S477N, RBD-E484K, RBD-N501Y and RBD-501Y.V2 have been deposited in the PDB with accession codes of 7EJY, 7EJZ, 7F6Y, 7EK0, and 7F6Z, respectively.
