# Supplementary material for: Structure-based analyses of neutralization antibodies interacting with naturally occurring SARS-CoV-2 RBD variants
Source: Cell Res. 2021 Sep 3;31(10):1126–9. doi: 10.1038/s41422-021-00554-1 (PMC8413711; doi:10.1038/s41422-021-00554-1)
Supplement: Supplementary file 1 — Supplementary Information [file 41422_2021_554_MOESM1_ESM.pdf]

SUPPLEMENTARY INFORMATION

Structure-based Analyses of Neutralization Antibodies Interacting with Naturally Occurring SARS-CoV-2 RBD Variants

Hua Xu[1], Bo Wang[1], Tian-ning Zhao[1], Zi-teng Liang[2], Tian-bo Peng[1], Xiao-hui Song[1], Jia-jing Wu[2], You-chun Wang[2], Xiao-dong Su[1]

[1]State Key Laboratory of Protein and Plant Gene Research, School of Life Sciences, and Biomedical Pioneering Innovation Center (BIOPIC), Peking University, Beijing, China;
[2]Division of HIV/AIDS and Sex-transmitted Virus Vaccines, Institute for Biological Product Control, National Institutes for Food and Drug Control (NIFDC) and WHO Collaborating Center for Standardization and Evaluation of Biologicals, Beijing, China.

These authors contribute equally: Hua Xu, Bo Wang, Tian-ning Zhao, Zi-teng Liang.

Correspondence: You-chun Wang (wangyc@nifdc.org.cn), Xiao-dong Su (xdsu@pku.edu.cn).

Materials and Methods

Mutational abundance analysis

Spike protein sequences were obtained from GISAID[1,2] and NCBI[3] databases. Metadata was from 2019nCoVR database developed and maintained by CNCB-NGDC[4]. To remove redundancies and filter sequences, SeqKit[5] was used to parse sequence ids, and filter sequences according to the metadata. We use MAFFT 7[6] to align all other sequences to the reference[7] along with the '--auto' option to identify mutation. Abundance and frequency were calculated using Python packages pandas[8] and NumPy[9] and visualized using matplotlib[10] and seaborn. R package UpSetR[11] was used to generate UpSet[12] plots.

Protein expression and purification

The receptor binding domain, as well as its mutants, of SARS-CoV-2 spike protein (residue 319-541) were cloned into pcDNA3.1 vector with an IL2 signal peptide at the N-terminal and a C-terminal 8×His-tag. Plasmids were transfected into HEK293F cells (cell density between 1~1.5 million cells/mL) using polyethylenimine (Polysciences). The conditioned media were collected after four days, and the proteins were purified using a Ni-NTA affinity column (GE Life Sciences), and Superdex 200 column (GE Life Sciences) in the final buffer: 20 mM HEPES, pH 7.2, 150 mM NaCl. For crystallization, the SARS-CoV-2 RBD and its mutants with an N-terminal 6×His-tag were cloned into pFastBac vector with a gp67 signal peptide, then bacmids were generated by using the Bac-to-Bac system (Invitrogen), SF-21 insect cells were then used to generate baculoviruses. Hi-5 insect cells were used to express proteins infected by recombinant baculoviruses when cell density reached 1.8 million cells/mL. The conditioned media were collected after 48 hours, concentrated and exchanged into 25 mM Tris, pH 8.0, 150 mM NaCl, then the proteins were purified as previously described.

The Fabs of BD-218, BD503, BD-508, BD-515, BD-604 and BD-623 were expressed using HEK293F cells. The heavy chains and light chains were cloned respectively into pcDNA3.1 vector with signal peptide and a C-terminal 6×His-tag. Plasmids of the heavy chain and light chain were admixed at a 1:1 ratio and then transfected into HEK293F cells by using polyethylenimine. The conditioned media were collected after 4 days, concentrated and exchanged into the binding buffer contained 25 mM Tris, pH 8.0, 150 mM NaCl. Then proteins were purified by Ni-NTA affinity column, and by gel filtration column Superdex 200 into the final buffer. The BD-368-2 Fab was obtained as previously described[13].

Surface plasmon resonance

A Biacore T200 (GE Healthcare, USA) was used to measure and compare the dissociation coefficients between antibodies and SARS-CoV-2 RBD as well as its mutants. Fabs of the antibodies were captured to 200~300 RU on a Series S Sensor

CM5 Chip (GE Healthcare). Then serial dilutions of SARS-CoV-2 RBD and mutants were injected, with concentrations from 20 to 0.63 nM (2-fold dilutions). All proteins were exchanged into running buffer containing 10 mM HEPES, pH 7.4, 150 mM NaCl, 3 mM EDTA and 0.005% (v/v) P20. The final data were processed by the Biacore Evaluation Software and fit to a 1:1 binding model.

Crystallization

The complexes of Fabs with SARS-CoV-2 RBD or the mutants were obtained by mixing the proteins at equimolar ratios and incubated at 4 °C for one hour, the complexes were then purified by Superdex 200 column in the final buffer. Purified complexes were concentrated to 10 mg/ml, crystallization then performed using the sitting-drop vapor diffusion method at 18 °C.

Crystals were obtained after 24 hours in the following conditions:

BD-503/RBD: 0.15 M Ammonium sulfate, 0.1 M Tris, pH 8.0, and 15% (w/v) PEG 4000;

BD-503/RBD-S477N: 0.1 M Magnesium chloride hexahydrate, 0.1 M Sodium citrate, pH 5.0, and 15% (w/v) PEG 4000;

BD-503/RBD-E484K: 0.1 M Magnesium chloride hexahydrate, 0.1 M Sodium citrate, pH 5.0, and 15% (w/v) PEG 4000;

BD-503/RBD-N501Y: 0.1 M Sodium citrate, pH 5.5, and 15% (w/v) PEG 6000;

BD-503/RBD-501Y.V2: 0.2 M Ammonium sulfate, 0.1 M Tris, pH 8.5, 12% (w/v) PEG 8000.

Data collection and structure determination

All crystals were picked by cryo-loops and placed in the corresponding reservoir solution, containing 20% (v/v) glycerol before flash-frozen in liquid nitrogen. All data were collected at beamline BL19U of the National Facility for Protein Science Shanghai, beamline BL17U of the Shanghai Synchrotron Radiation Facility (SSRF) and beamline BL1A of the KEK Photon Factory. The data were integrated and scaled using HKL2000 (HKL Research, USA). Further calculation was performed using molecular replacement in the suite Phenix[13]. All structural models were refined using Phenix and manually corrected in Coot[14].

Pseudovirus neutralization assay

The pseudovirus neutralization assays were performed using Huh-7 cell lines. Pseudovirus were prepared as previously described[16]. Various concentrations of antibodies (3-fold serial dilution using DMEM) were mixed with the same volume of SARS-CoV-2 pseudovirus in a 96 well-plate. The mixture was incubated for 1 h at 37 °C and supplied with 5% CO_2. Pre-mixed Huh-7 cells were added to all wells and incubated for 24 h at 37 °C and supplied with 5% CO_2. After incubation, the supernatants were removed, and D-luciferin reagent (Invitrogen) was added to each well and measured luciferase activity using a microplate spectrophotometer (PerkinElmer EnSight). The inhibition rate is calculated by comparing the OD value to the negative and positive control wells. IC_{50} were determined by a four-parameter

logistic regression using GraphPad Prism 8.0 (GraphPad Software Inc.).

Supplementary Figures

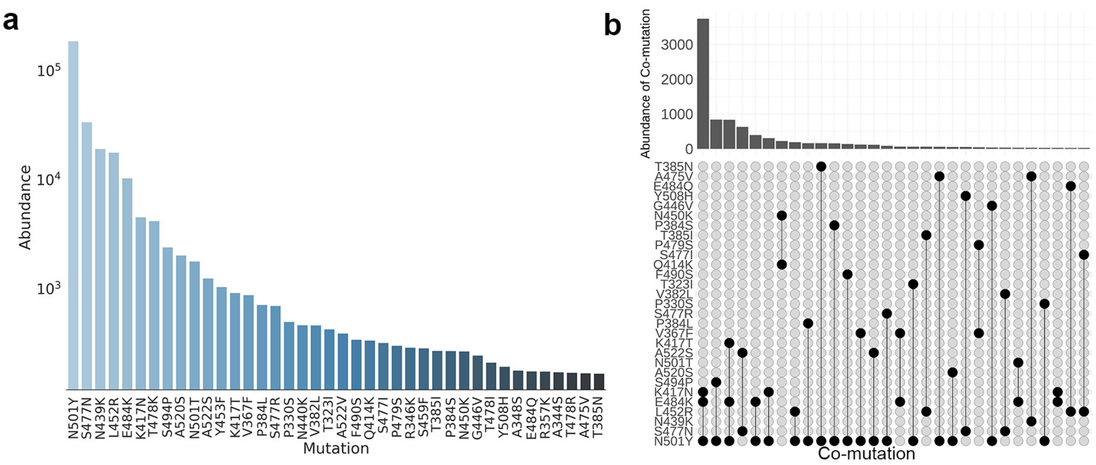

Fig. S1. Abundance of SRAS-CoV-2 RBD mutations and co-mutations.

a Abundance of mutations in the SARS-CoV-2 RBD. **b** Th co-mutational variety of SARS-CoV-2 RBD and their abundance.

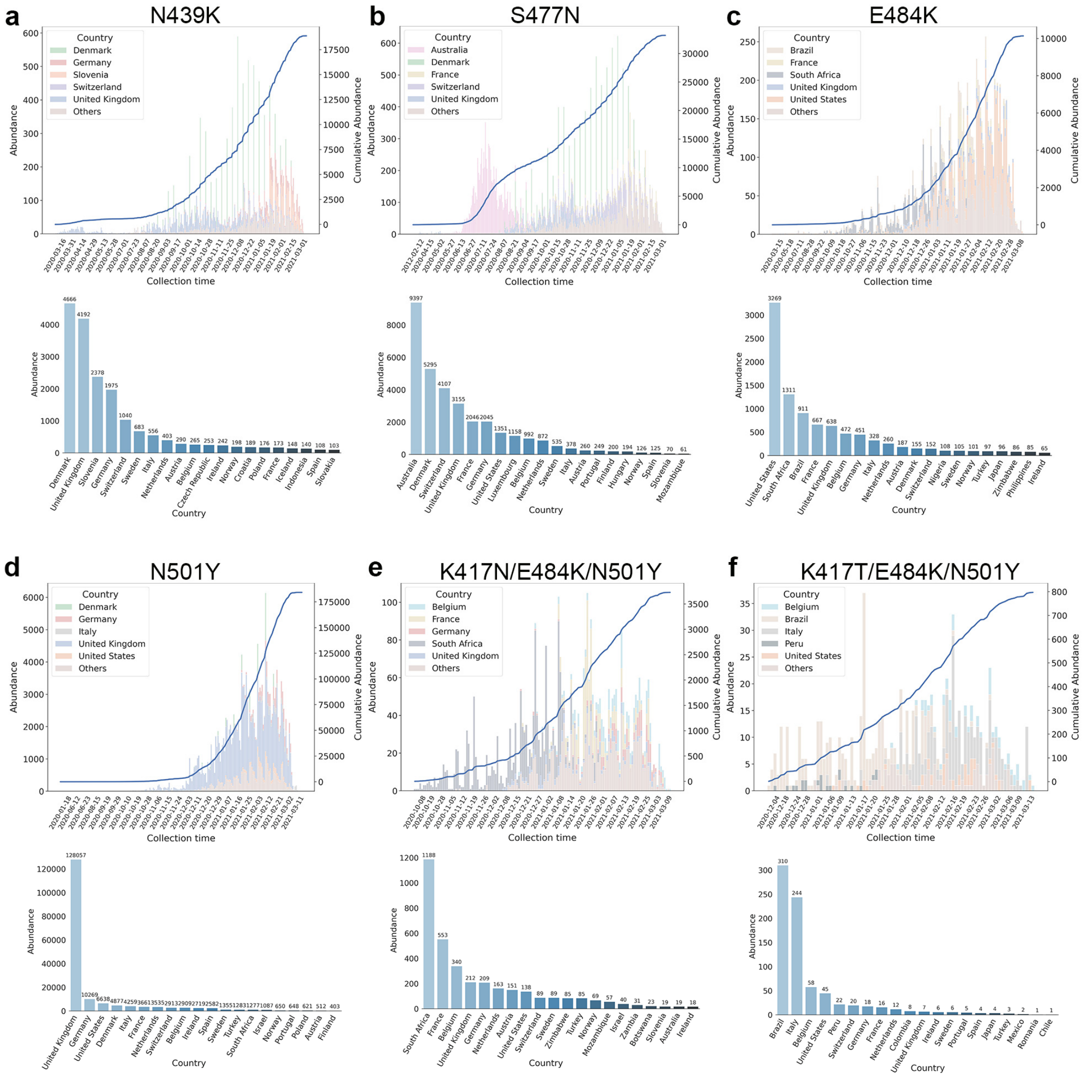

Fig. S2. Analysis of common naturally occurring SARS-CoV-2 RBD mutations.

a-f The mutational abundance in terms of time scale and regional distribution of N439K, S477N, E484K, N501Y, K417N/E484K/N501Y (501Y.V2) and K417T/E484K/N501Y (501Y.V3).

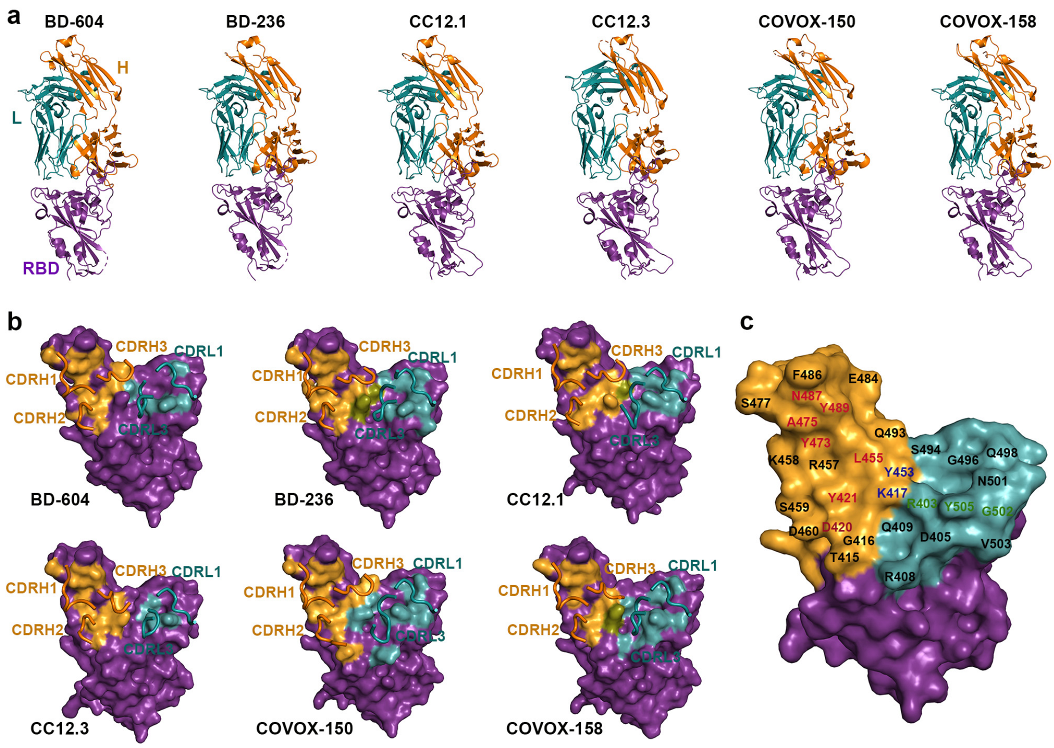

Fig. S3. Epitopes of VH3-53/3-66 derived public antibodies.

a Crystal structures of SARS-CoV-2 RBD in complex with other VH3-53/3-66 derived antibodies: BD-604 (PDB: 7CH4), BD-236 (PDB: 7CHB), CC12.1 (PDB: 6XC2), CC12.3 (PDB: 6XC4), COVOX-150 (PDB: 7BEI), and COVOX-158 (PDB: 7BEK). **b** Epitopes of BD-604, BD-236, CC12.1, CC12.3, COVOX-150 and COVOX-158. The RBD is shown in a surface view. **c** Position of residues on RBD surface that recognized by VH3-53/3-66 derived antibodies. Residues on orange surface: can be recognized by VH domains of antibodies; cyan surface: can be recognized by VL domains of antibodies. Residues labeled in red: can be recognized by VH domains of most VH3-53/3-66 antibodies; green: can be recognized by VL domains of most VH3-53/3-66 antibodies; blue: can be recognized by VH domains (CC12.1 and CC12.3) or VL domains (BD-604, COVOX-150 and COVOX-158) or both VH and VL domains (BD-236, CC12.1 and COVOX-158) of the germline-based antibodies.

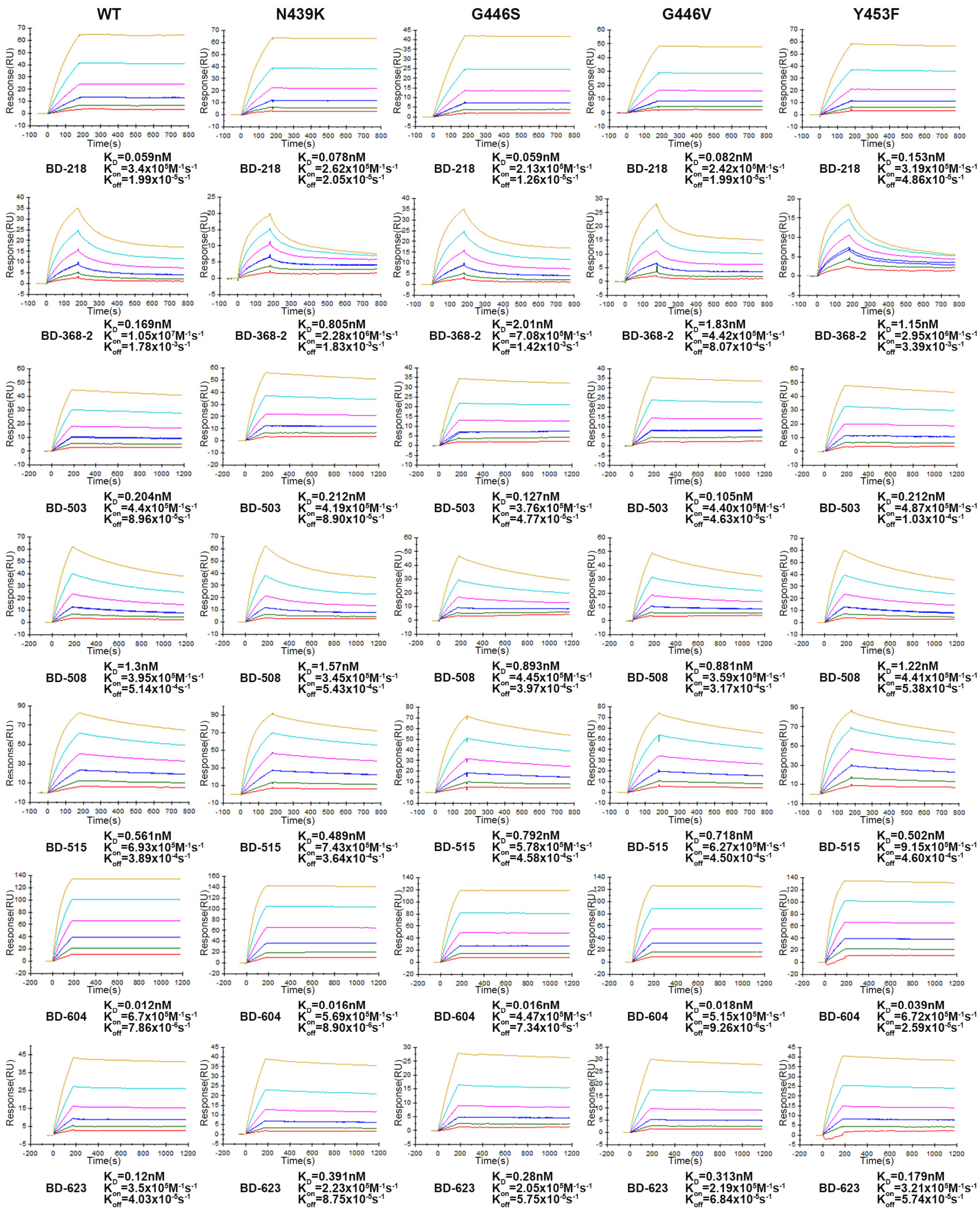

Fig. S4. Surface plasmon resonance sensorgrams of neutralizing antibodies binding to RBD and RBD variants N439K, G446S, G446V and Y453F

Surface plasmon resonance sensorgrams of BD-218, BD-368-2, BD-503, BD-508, BD-515, BD-604 and BD-623 binding to RBD and its variants N439K, G446S, G446V and Y453F. All analyses were performed by using a serial 2-fold dilution of purified RBDs as the analyte, starting from 20 nM to 0.625 nM.

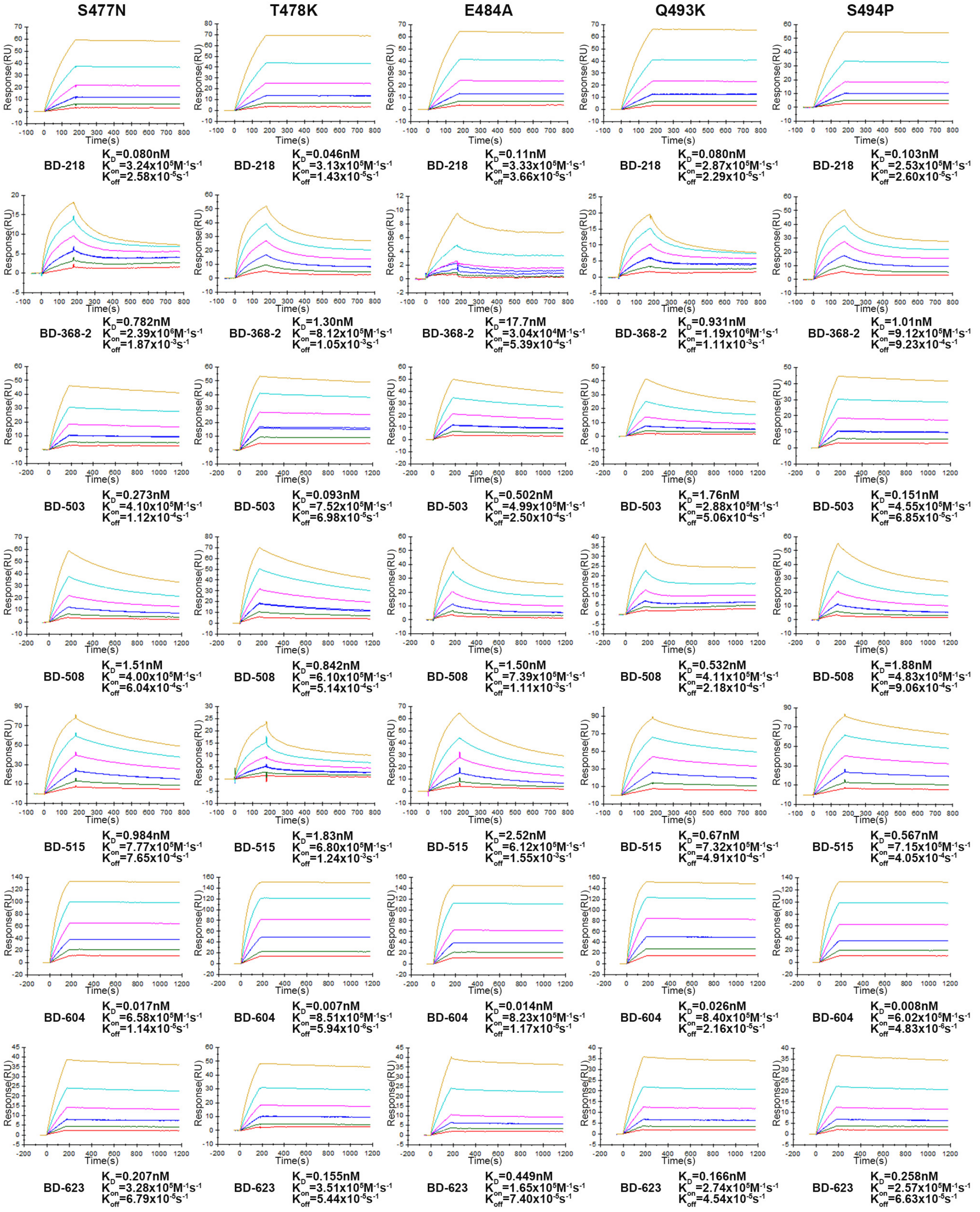

Fig. S5. Surface plasmon resonance sensorgrams of neutralizing antibodies binding to RBD variants S477N, T478K, E484A, Q493K and S494P.

Surface plasmon resonance sensorgrams of BD-218, BD-368-2, BD-503, BD-508, BD-515, BD-604 and BD-623 binding to RBD variants S477N, T478K, E484A, Q493K and S494P. All analyses were performed by using a serial 2-fold dilution of purified RBDs as the analyte, starting from 20 nM to 0.625 nM.

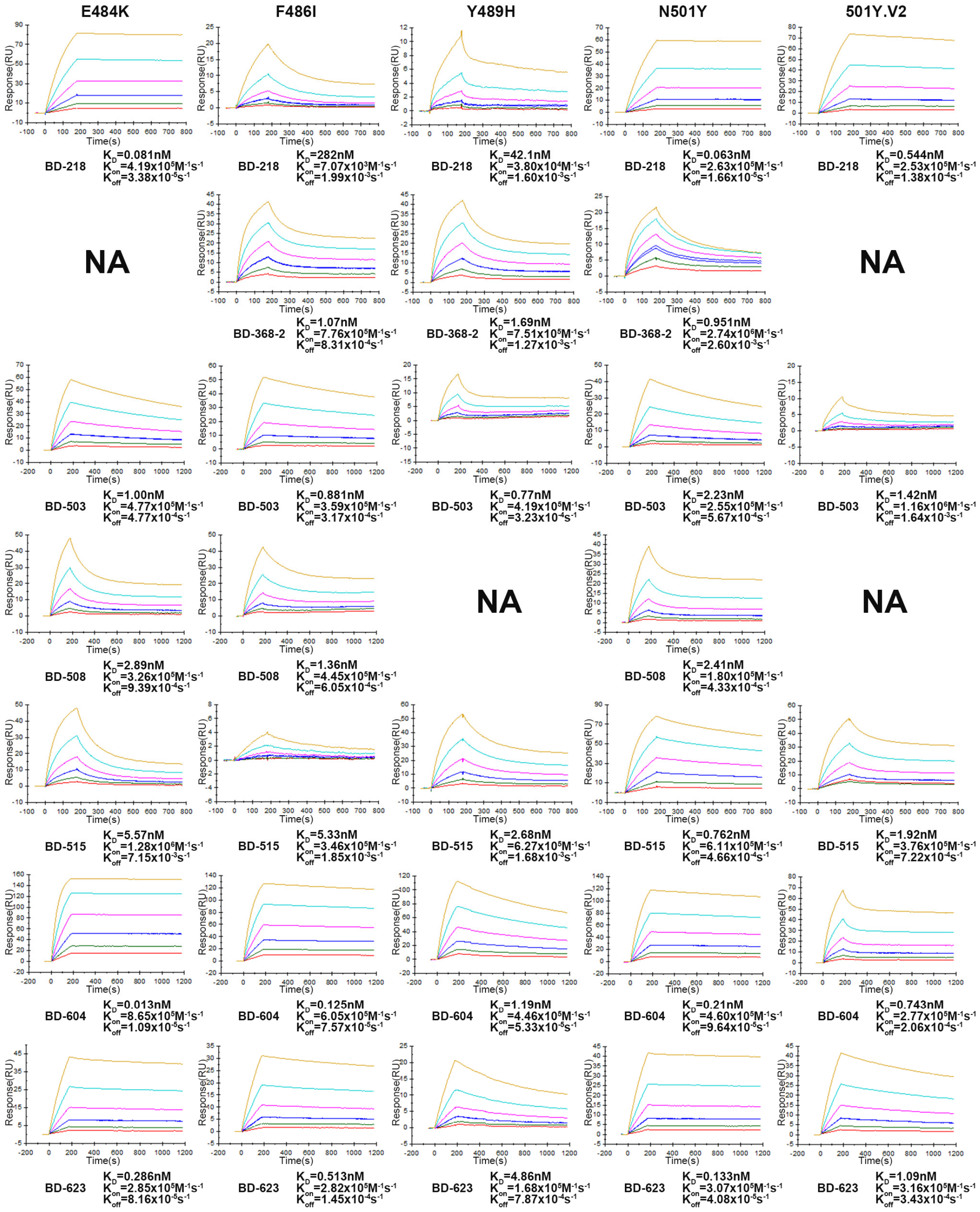

Fig. S6. Surface plasmon resonance sensorgrams of neutralizing antibodies binding to RBD variants E484K, F486I, Y489H, N501Y and 501Y.V2.

Surface plasmon resonance sensorgrams of BD-218, BD-368-2, BD-503, BD-508, BD-515, BD-604 and BD-623 binding to RBD variants E484K, F486I, Y489H, N501Y and 501Y.V2. NA: not acquired. All analyses were performed by using a serial 2-fold dilution of purified RBDs as the analyte, starting from 20 nM to 0.625 nM.

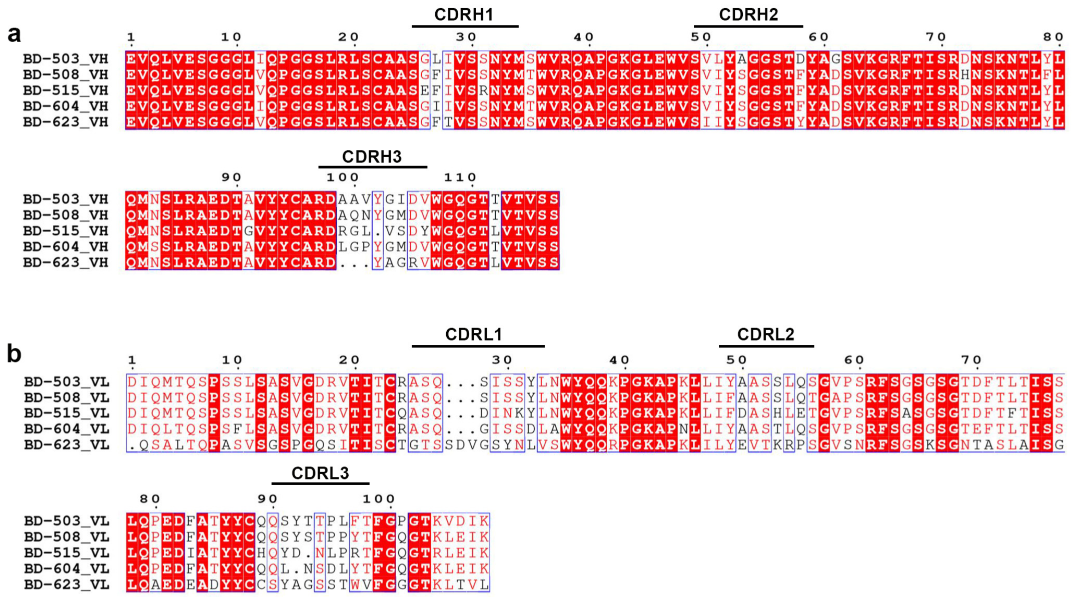

Fig. S7. Sequence alignments of VH3-53/3-66 derived public antibodies.

Sequence alignments of VH3-53/3-66 derived public antibodies in VH domains (**a**) and VL domains (**b**). The germline-based public antibody showed high identity in VH domain with a shorter CDRH3 compared to other antibodies.

a

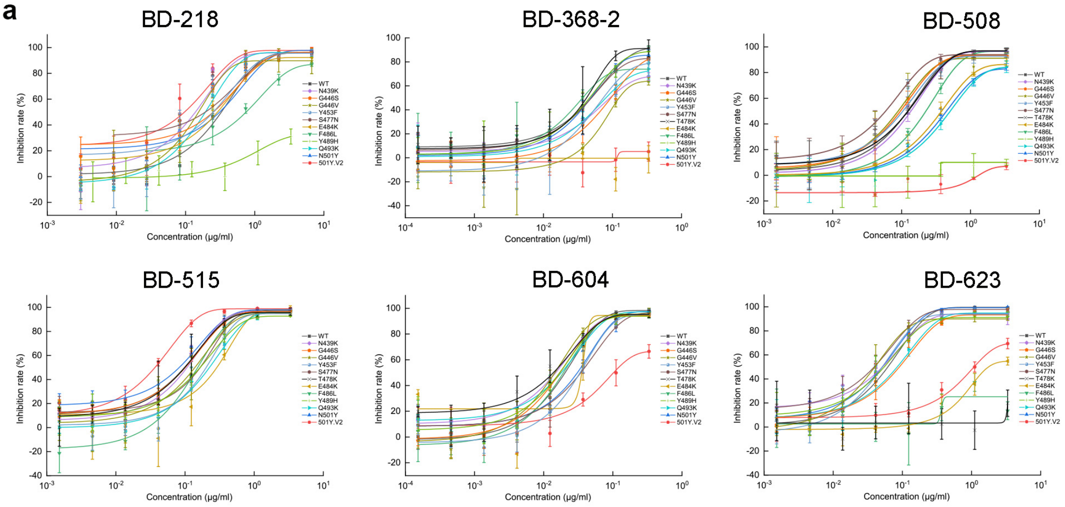

b

mAbs	EC$_{50}$ of mAbs to SARS-CoV-2 variants												
	WT	N439K	G446S	G446V	Y453F	S477N	T478K	E484K	F486L	Y489H	Q493K	N501Y	501Y.V2
**BD-218**	0.376	0.152	0.131	0.171	0.170	0.156	-	0.239	1.072	>3.333	0.210	0.133	0.048
**BD-368-2**	0.032	0.071	0.096	0.282	0.079	0.039	0.036	>0.333	0.035	0.031	0.075	0.044	>0.333
**BD-508**	0.098	0.114	0.069	0.079	0.090	0.063	0.088	0.349	0.197	>3.333	0.603	0.450	>3.333
**BD-515**	0.071	0.073	0.076	0.139	0.192	0.096	0.066	0.265	0.237	0.121	0.195	0.054	0.029
**BD-604**	0.020	0.012	0.021	0.015	0.031	0.024	0.009	0.024	0.021	0.014	0.014	0.019	0.088
**BD-623**	0.052	0.034	0.074	0.049	0.050	0.036	>3.333	2.002	>3.333	0.043	0.075	0.040	0.718

Fig. S8. Neutralizing abilities of NAbs.

a Neutralization potency of BD-218, BD-368-2, BD-508, BD-515, BD-604, and BD-623 to SARS-CoV-2 S protein variants measured by pseudovirus neutralization assay. Data are represented as mean ± SD. IC$_{50}$ was calculated by fitting a four-parameter logistic curve. **b** EC$_{50}$s of the NAbs to pseudovirus carrying SARS-CoV-2 S protein variants. Red indicates major fold-change that the EC$_{50}$ cannot obtained from the assay.

Table S1. Statistics of X-ray data collection and refinement.

	7EJY	7EJZ	7F6Y	7EK0	7F6Z
**Wavelength**	0.9785	0.9785	1.1	0.9785	1.1
**Resolution range**	29-3.04 (3.14-3.04)	41.66-3.63 (3.76-3.63)	20.0-3.0 (3.11-3.0)	42.54-2.70(2.8-2.70)	20.0-3.0 (3.11-3.0)
**Space Group**	C 2 2 21	C 2 2 21	C 2 2 21	C 2 2 21	C 2 2 21
**Unit cell**	80.432 144.083 146.657	80.711 145.915 146.789	80.28 149.07 146.69	81.426 149.665 146.433	82.92 146.84 145.65
	90 90 90	90 90 90	90 90 90	90 90 90	90 90 90
**Total reflections**	203847 (17030)	12896 (12840)	122052 (12440)	12896 (12840)	110212 (10973)
**Unique reflections**	12896 (12840)	10003 (930)	17918 (1762)	23686 (1595)	18090 (1777)
**Multiplicity**	12.5 (11.0)	12.8 (12.9)	6.8 (7.1)	13.1 (13.6)	6.1 (6.2)
**Completeness (%)**	92.73 (56.95)	95.96 (81.45)	99.49 (99.94)	94.68 (65.37)	99.45 (99.72)
**Mean I/sigma (I)**	10.61 (2.00)	7.96 (2.71)	8.36 (2.12)	16.64 (1.84)	8.33 (1.50)
**Wilson B-factor**	67.35	67.35	54.64	41.80	68.32
**R-merge**	0.2638 (0.8767)	0.4503 (1.005)	0.20 (0.76)	0.232 (1.22)	0.20 (1.00)
**R-meas**	0.275 (0.9198)	0.4691 (1.047)	0.22 (0.82)	0.2417 (1.268)	0.22 (1.10)
**R-pim**	0.07652 (0.2718)	0.1301 (0.2923)	0.08 (0.30)	0.06685 (0.3411)	0.09 (0.43)
**CC 1/2**	0.971 (0.783)	0.958 (0.862)	0.99 (0.82)	0.991 (0.799)	0.99 (0.90)
**Reflections used in refinement**	15550 (934)	9689 (808)	17910 (1761)	23562 (1595)	18080 (1776)
**Reflections used for R-free**	1552 (93)	966 (88)	1782 (162)	1976 (138)	1792 (175)
**R-work**	0.2113 (0.2686)	0.2468 (0.4440)	0.20 (0.32)	0.2161 (0.2874)	0.21 (0.34)
**R-free**	0.2501 (0.3461)	0.2872 (0.5028)	0.25 (0.38)	0.2872 (0.5028)	0.25 (0.37)
**Number of non-hydrogen atoms**	4757	4774	4757	4747	4780
**Macromolecules**	4743	4760	4743	4746	4752
**Ligands**	14	14	14	1	28
**Protein residues**	622	624	622	621	624
**RMS (bonds)**	0.014	0.009	0.01	0.012	0.01
**RMS (angles)**	1.72	1.40	1.18	1.41	1.35
**Ramachandran favored (%)**	93.15	87.48	93.96	93.64	93.16
**Ramachandran allowed (%)**	6.53	11.87	5.71	6.04	6.68
**Ramachandran outliers (%)**	0.33	0.65	0.33	0.33	0.16
**Average B-factor**	52.98	70.92	44.17	44.79	61.81

REFERENCES

1. Elbe S. & Buckland-Merrett G. Data, disease and diplomacy: GISAID's innovative contribution to global health[J]. *Global Challenges* **1**(1): 33-46 (2017).
2. Shu Y. & McCauley J. GISAID: Global initiative on sharing all influenza data–from vision to reality[J]. *Eurosurveillance* **22**(13): 30494 (2017).
3. Coordinators, N.R. Database resources of the National Center for Biotechnology Information. *Nucleic Acids Research* **44**, D7-D19 (2016).
4. Song, S. et al. The global landscape of SARS-CoV-2 genomes, variants, and haplotypes in 2019nCoVR. *Genomics, Proteomics & Bioinformatics* (2020).
5. Shen, W., Le, S., Li, Y. & Hu, F. SeqKit: a cross-platform and ultrafast toolkit for FASTA/Q file manipulation. *PloS one* **11**(10), e0163962 (2016).
6. Katoh, K. & Standley, D. M. MAFFT multiple sequence alignment software version 7: improvements in performance and usability. *Molecular biology and evolution* **30**(4), 772-780 (2013).
7. Katoh, K. & Frith, M.C. Adding unaligned sequences into an existing alignment using MAFFT and LAST. *Bioinformatics* **28**(23), 3144-3146 (2012).
8. McKinney W. Data structures for statistical computing in python. *Proceedings of the 9th Python in Science Conference* **445**, 51-56 (2010).
9. Harris, C.R. et al. Array programming with NumPy. *Nature* **585**, 357-362 (2020).
10. Hunter, John D. Matplotlib: A 2D graphics environment. *IEEE Annals of the History of Computing* **9**(03), 90-95 (2007).
11. Conway, J.R. Lex, A. & Gehlenborg, N. UpSetR: an R package for the visualization of intersecting sets and their properties. *Bioinformatics* **33**, 2938-2940 (2017).
12. Lex, A. Gehlenborg, N. Strobelt, H. Vuillemot, R. & Pfister, H. UpSet: Visualization of Intersecting Sets. *IEEE Transactions on Visualization and Computer Graphics* **20**, 1983-1992 (2014).
13. Du, S. et al. Structurally Resolved SARS-CoV-2 Antibody Shows High Efficacy in Severely Infected Hamsters and Provides a Potent Cocktail Pairing Strategy. *Cell* **183**, 1013-1023 e1013 (2020).
14. Liebschner, D. et al. Macromolecular structure determination using X-rays, neutrons and electrons: recent developments in Phenix. *Acta Crystallographica Section D* **75**, 861-877 (2019).
15. Emsley, P. Lohkamp, B. Scott, W.G. & Cowtan, K. Features and development of Coot. *Acta Crystallographica Section D* ***66***, 486-501 (2010).
16. Cao, Y. et al. Humoral immune response to circulating SARS-CoV-2 variants elicited by inactivated and RBD-subunit vaccines. *Cell research* (2021). https://doi.org/10.1038/s41422-021-00514-9
